# Effect of Machining Parameters and Tool Wear on Surface Uniformity in Micro-Milling

**DOI:** 10.3390/mi9060268

**Published:** 2018-05-29

**Authors:** Zhanwen Sun, Suet To

**Affiliations:** State Key Laboratory in Ultra-Precision Machining Technology, Department of Industrial and Systems Engineering, The Hong Kong Polytechnic University, Hung Hom, Kowloon, Hong Kong SAR, China; 16901527r@connect.polyu.hk

**Keywords:** cutting mechanism, micro-milling, ploughing effect, surface uniformity

## Abstract

In micro-milling, the periodically varying chip thickness, which varies with tool rotation, leads to varying degrees of minimum chip thickness effect and ploughing effect during surface generation. This results in a change of roughness in the cross-sectional direction of the micro-grooves, giving a non-uniform surface quality. However, the factors influencing surface uniformity in micro-milling are not fully understood. In the present work, the effect of the machining parameters and tool wear on surface uniformity in micro-milling is theoretically and experimentally studied. A mathematical model is proposed to predict the varying surface roughness in the cross-sectional direction of the micro-grooves, which is experimentally validated by fabricating a set of 800 µm wide micro-grooves. The theoretical and experimental results reveal that, compared to the normally adopted Ra or Sa, the relative standard deviation of roughness (RSDS) is more appropriate to evaluating surface uniformity. When machining under small feed rates and small cutting depths, the surface uniformity deteriorates as the feed rate increases and improves as the cutting depth increases. The blunt cutting edge induced by tool wear enhances the surface uniformity and increases the surface roughness at the same time. This research furthers understanding of the various cutting mechanisms in micro-milling and can be applied to the optimization of machining parameters in micro-milling.

## 1. Introduction

Micro-devices have become increasingly popular in industries, such as aerospace, biomedical and microelectronics, due to their many advantages, such as their compactness, low energy consumption and material saving [1,2]. Moreover, the widespread application of micro-devices can also be attributed to the fast-emerging micro-machining technologies, such as *X*-ray lithography electro-deposition molding (LIGA) [3], laser machining [4,5], and tool-based micro mechanical machining technologies [6]. However, the non-conventional manufacturing technologies have some common drawbacks, such as limited workpiece material, excessive cost, simple micro-scale features and low efficiency [7]. Because of its capacity to fabricate complex 3D structures using a variety of materials (metallic and nonmetallic), micro-milling technology has received extensive attention from researchers and designers.

As the chip load and tool edge radius of micro-milling machines normally have the same size order of magnitude, the micro-milling process is dominated by special cutting mechanisms, such as minimum chip thickness effect [8], ploughing effect [9] and size effect [10]. Furthermore, due to the variation of the relative tool sharpness (RTS) induced by the chip thickness, which varies with tool rotation, the dominant cutting mechanisms in micro-milling also change as the tool rotates [11]. For instance, Ventura et al. [12] reported that the amplitude of cutting force components increased as the RTS decreased, which could lead to high surface roughness. The relationship between RTS and the material removal mechanism with respect to Mg and Cu alloy was also studied by Rahman et al. [13], who concluded that the material removal mechanism gradually transitions from shearing to ploughing as RTS decreases. In micro-milling, the cutting mechanisms, which vary with tool rotation, inevitably lead to a non-uniform surface quality in the cross-sectional direction of the micro-grooves. However, there has been very little research on the development of mathematical models for estimating the varying roughness.

Some scholars have conducted preliminary experimental examinations of the variation of the cutting mechanisms in micro-milling and its induced non-uniform surface quality. Ramos et al. reported [14] that the surface roughness of micro-milled grooves changes in the cross-sectional direction. They explained that this non-uniform surface quality is caused by a transformation of the machining mechanisms involved in ploughing and shearing. Fernando et al. [8] validated the cutting mechanisms, which vary with tool rotation, in the micro-milling of steel by observing the differences in surface topographies between the groove center and the sides. The non-uniform surface quality of micro-milling has also been observed in brittle materials [15]. For example, by conducting the micro-milling experiments on glass, Arif et al. [16] concluded that brittle fractures tend to occur in the groove centers where the instantaneous chip thickness reaches and exceeds a critical value. However, even though the non-uniform surfaces generated by micro-milling were previously investigated using experimental results, the effects of machining parameters and tool wear on surface uniformity are still not fully understood.

In the present study, a mathematical model considering the variation of RTS is proposed to estimate the change in the surface roughness of the cross-sectional direction of the micro-grooves. By adopting a method, named the relative standard deviation of roughness, to evaluate the surface uniformity, the effect on the surface uniformity of machining parameters, including cutting speed, feed rate and depth of cut, as well as tool wear, is discussed. The results of this study further understanding of the various cutting mechanisms in micro-milling and can be used to optimize machining processes to produce a high-uniform surface quality.

## 2. Experimental Setup

**Figure 1:**
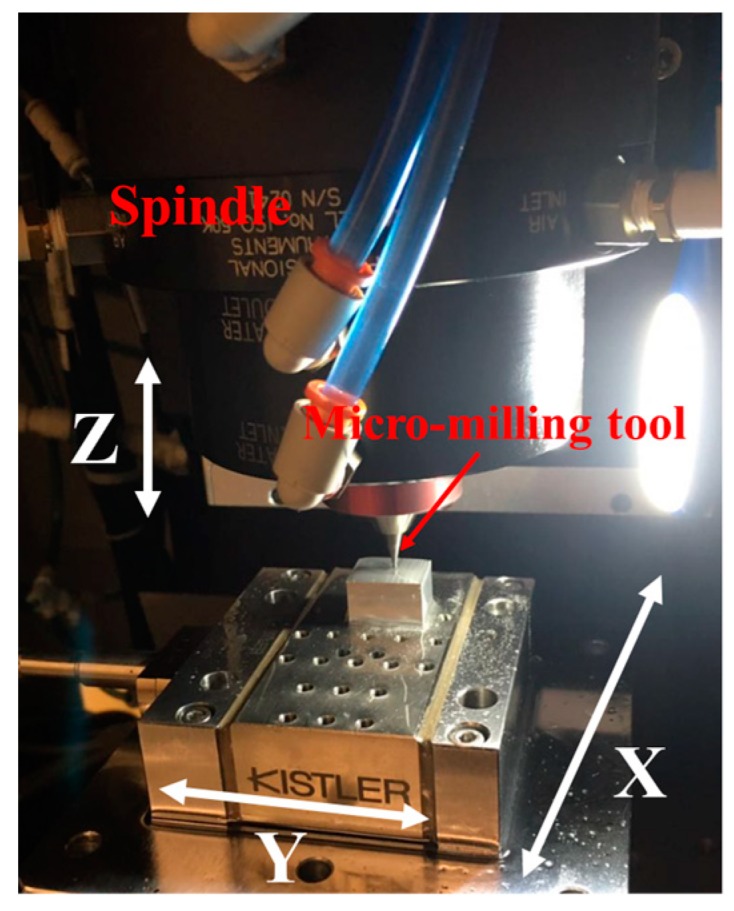
Schematic of the micro-milling machine and milling methodology.

A set of full-immersion micro-milling experiments were conducted on a 3-axes high-precision computer numerical control (CNC) micro-milling machine from Aerotech Company (Pittsburgh, PA, USA), as shown in Figure 1. The movement distance of the X and Y slides was 300 mm, with its resolution and position accuracy reaching 0.01 µm and 0.1 µm, respectively. A two-flute carbide micro-milling tool was fixed on the high-speed spindle and rotated to achieve the rotational cutting trajectory. The spindle, in turn, was mounted on the Z slide of the machine tool in a vertical position. Micro-milling tools of diameter 800 µm were used in the experiments. The tool edge radius was measured as 1.2 µm.

The workpiece material adopted in the experiments was Aluminum 6061. In the machining process, the workpiece was carried by the X and Y slides moving horizontally, and the spindle was driven by the Z slide moving vertically to achieve a fabrication with different cutting depths. This allowed a set of parallel micro-grooves to be machined, with a cutting length of 10 mm and a cross-distance between two neighboring micro-grooves of 4 mm. Three groups of machining experiments were conducted to study the effect of machining parameters on the surface uniformity, as shown in Table 1. To enhance the reliability of the experiment results, two repeated experiments were conducted for each group, and the average values of the experiment data were used to analyze the effect of machining parameters on surface uniformity. The machining conditions are shown in Table 2.

The 3D topographies of the bottom surfaces of the micro-grooves were acquired using an Optical Surface Profiler (Zygo Corporation, Middlefield, CT, USA). Additionally, a Hitachi TM3000 (Hitachi High-Technologies Corporation, Tokyo, Japan) scanning electron microscope (SEM) was used to observe the surface topography. A non-contact 3-dimensional optical device based on focus variation (Alicona IFM G4, Alicona Imaging GmbH, Raaba bei Graz, Austria) was employed to measure the 3D topography of the cutting edge.

## 3. Mathematical Model for Non-Uniform Roughness

In micro-milling the micro-grooves, the chip thickness increased from zero, at the tool-workpiece engagement point, to a maximum value at the groove center, and then back to zero again at the tool-workpiece disengagement point. Regarding the minimum chip thickness and ploughing effect, it is commonly known that the periodically varying chip thickness leads to a change in the roughness between the groove sides and the center. In this section, a mathematical model is introduced to simulate the varying surface roughness in the cross-sectional direction of the micro-grooves.

**Figure 2:**
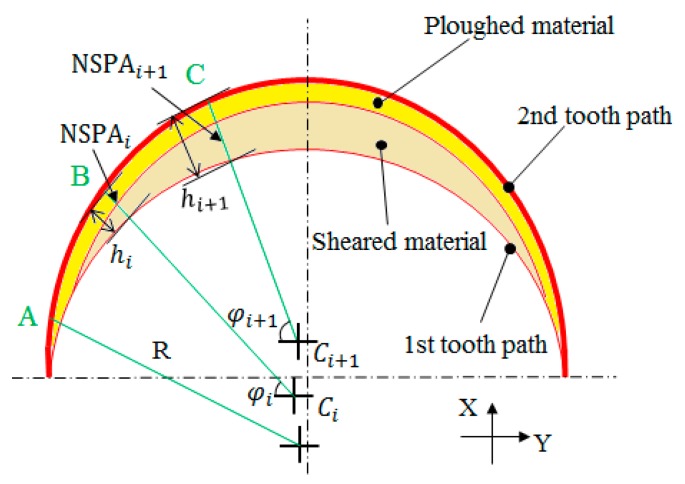
Model for the calculation of ${\mathrm{NSPA}}_{i}$.

As the varying roughness mainly results from the minimum chip thickness effect and the level of the ploughing effect, varying with the change of the chip thickness, the stochastic roughness $Ra_{s}^{i}$ at the rotation angle $\varphi_{i}$ can be estimated as

(1) $$Ra_{s}^{i}=k{\left({\mathrm{NSPA}}_{i}\right)}^{n}$$

where *k* and *n* are material related coefficients and the ${\mathrm{NSPA}}_{i}$ is the variation of the normal specific ploughing amount, which is defined as the arithmetic product between the ratio of the volume of ploughed material to the volume of total tool-workpiece contact material and the normal vector of chip flow at the rotation angle $\varphi_{i}$, as illustrated in Figure 2. It noteworthy that the stochastic roughness $Ra_{s}^{i}$ calculated in Equation (1) is not equivalent to the surface roughness *Ra*. $Ra_{s}^{i}$ only refers to the roughness induced by the ploughing effect. To calculate different $Ra_{s}^{i}$ values at different rotation angles $\varphi_{i}$, an index, named the normal specific ploughing amount (${\mathrm{NSPA}}_{i}$), was used to evaluate the ploughing effect. ${\mathrm{NSPA}}_{i}$ is derived from the specific ploughing amount (SPA) [17,18], which is generally used as an index for ploughing, by incorporating the relative tool sharpness. ${\mathrm{NSPA}}_{i}$ can be expressed by:

(2) $${\mathrm{NSPA}}_{i}=\;\frac{A_{p}^{i}\times \sin\gamma_{i}}{A_{t}^{i}}=\;\frac{A_{p}^{i}\times \sin\gamma_{i}}{A_{s}^{i}+A_{p}^{i}}$$

where $A_{t}^{i}$ represents the total volume of the tool-workpiece contact material, and $A_{s}^{i}$ and $A_{p}^{i}$ are the volumes of the sheared and ploughed material, respectively. $\gamma_{i}$ is the negative rake angle.

To calculate ${\mathrm{NSPA}}_{i}$ a coordinate system is defined, with its original point at the rotation center of the micro-milling tool. For a two-flute micro-milling tool, the coordinates of the cutting trajectory ($x_{c}^{i}$, $y_{c}^{i}$) at ($\varphi_{i}$) can be expressed as:

(3) $$\{\begin{array}{l} x_{c}^{i}=\frac{f_{t}}{\pi }\varphi_{i}+r_{o}\sin\left(\varphi_{i}-\pi n_{f}\right)+Rsin\left(\varphi_{i}+\delta \right)\\ y_{c}^{i}=r_{o}\cos\left(\varphi_{i}-\pi n_{f}\right)+Rcos\left(\varphi_{i}+\delta \right)\;\;\;\;\;\;\;\;\;\;\;\;\;\;\; \end{array}$$

where $f_{t}$ is the feed per tooth, *R* is the normal Radius of the milling tool, *n* is the spindle rotational speed, $n_{f}$ equals 0 or 1, representing the ordinal number of the two cutting flutes, and $r_{o}$ and δ denote the tool runout length and runout angle respectively.

Based on Equation (3), the instantaneous uncut chip thickness ($h_{i}$) between two neighboring tool paths can be computed and simplified according to Reference [19] as:

(4) $$\begin{array}{l} h_{i}={\left(-1\right)}^{n_{f}}\times 2r_{o}[\frac{\sin\left(\delta \right)\sin\left(\varphi_{i}-\pi n_{f}\right)}{\pi R}-\cos\left(\delta \right)]\\ \;+f_{t}[\sin\left(\varphi_{i}-\pi n_{f}\right)\;-\frac{f_{t}}{\pi R}\sin\left(\varphi_{i}-\pi n_{f}\right)\cos\left(\varphi_{i}-\pi n_{f}\right)\\ \;+\frac{f_{t}}{\pi R}\cos^{2}\left(\varphi_{i}-\pi n_{f}\right)] \end{array}$$

As seen in Equation (4), the instantaneous chip thickness equals zero at the entrance and departure points of the tool, and reaches a maximum value when $\varphi_{i}-\pi n_{f}=\frac{\pi }{2}$. The maximum chip thickness $h_{\max}$ can be expressed as:

(5) $$h_{\max}=\;{\left(-1\right)}^{n_{f}}\times 2r_{o}[\frac{\sin\left(\delta \right)}{\pi R}-\cos\left(\delta \right)]+\;f_{t}$$

Thus, the degree of ploughing changes according to the varying chip thickness, and is highest at the groove sides and lower at the groove center, thereby resulting in a non-uniform surface quality. In addition, due to the minimum chip thickness effect, the fact that there are almost no chips formed at the groove sides makes the non-uniform phenomenon more pronounced.

**Figure 3:**
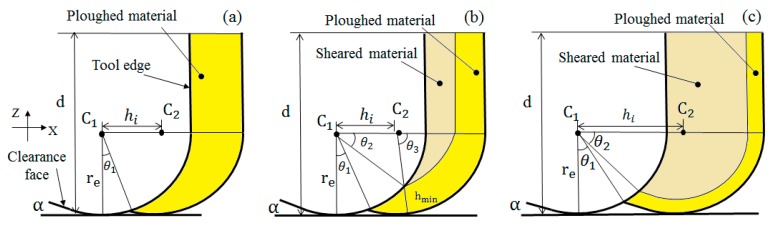
Geometry models of cutting regions at different uncut chip thicknesses with (**a**) a lower than minimum chip thickness; (**b**) a beyond minimum chip thickness without the influence of a clearance angle; and (**c**) an uncut chip thickness influenced by a clearance angle.

Considering the minimum chip thickness ($h_{\min}$) and tool edge clearance angle (α), three different intervals for each cutting flute can be found when $h_{\max}>\sqrt{{r_{e}}^{2}\sin^{2}\alpha +2r_{e}h_{\min}+{h_{\min}}^{2}}+r_{e}\sin\alpha$, as illustrated in Figure 3a–c. These intervals represent the corresponding points A, B and C in Figure 2 [17], where $r_{e}$ represents the tool edge radius. For Al6061, the minimum chip thickness is estimated to be $\sim 0.4r_{e}$ [20] and the elastic recovery coefficient ρ is estimated to be 0.09 [21].

For the point A, as schematically shown in Figure 3a, the uncut chip thickness is less than the minimum chip thickness, so no chips are generated at this point. Thus, the total tool-workpiece contact volume at A is classified as ploughed material. Based on Equation (2), ${\mathrm{NSPA}}_{i}$ at point A can be calculated as:

(6) $${\mathrm{NSPA}}_{i}=\sin\gamma_{i}\;\;\;\;\;\;\;(h_{i}<h_{\max})$$

The negative rake angle $-\gamma_{i}$ is given in Reference [22], using the average method, as:

(7) $$\gamma_{i}=\left[\left(1-\frac{h_{i}}{r_{e}}\right)\times \sin^{-1}\left(1-\frac{h_{i}}{r_{e}}\right)+\sqrt{1-{\left(1-\frac{h_{i}}{r_{e}}\right)}^{2}}-\frac{\pi }{2}\right]/\frac{h_{i}}{r_{e}}$$

where $h_{i}$ is the instantaneous chip thickness of the rotation angle $\varphi_{i}$.

As for point B, the instantaneous uncut chip thickness is larger than $h_{\min}$, but not large enough to reach the clearance angle. When $h_{i}<2r_{e}\sin\alpha$, the geometric profile of the cutting edge can be regarded as a perfect circle. Considering the influence of the ploughing effect from both the side wall and the bottom tool edge, the volume of the total tool cutting material ($A_{t}^{i}$) at an instantaneous uncut chip thickness ($h_{i}$) can be written as:

(8) $$A_{t}^{i}=r_{e}^{2}\times \theta_{1}+0.5h_{i}r_{e}\cos\theta_{1}+\left(d-r_{e}\right)h_{i}\;\;\;\;\;\;\;(h_{\min}<h_{i}<2r_{e}\sin\alpha )$$

with

(9) $$\theta_{1}=\{\begin{array}{c} \sin^{-1}(\frac{h_{i}}{2r_{e}})\;0<h_{i}<2r_{e}\sin\alpha \\ \frac{\pi }{2}-\alpha -\sin^{-1}\frac{\left[r_{e}\cos\alpha \;-\;\tan\alpha (h_{i}\;-\;r_{e}\sin\alpha )]\;\times \;\sin(\frac{\pi }{2}\;+\;\alpha \right)}{r_{e}}\;\;\;\;2r_{e}\sin\alpha \leq h_{i}\leq h_{\max} \end{array}$$

where *d* is the depth of cut. According to the geometric relation, the volume of ploughed material ($A_{p}^{i}$) at point B can be expressed as:

(10) $$\begin{array}{c} A_{p}^{i}=A_{t}^{i}-{\left(r_{e}-h_{\min}\right)}^{2}\times \theta_{3}+r_{e}^{2}\times \theta_{2}-\frac{h_{i}}{2}\left(r_{e}-h_{\min}\right)\sin\theta_{3}-\left(d-r_{e}\right)(h_{i}\\ -h_{\min})\;\;\;\;\;\;\;\;\;\;\;\;\;\;\;\;(h_{\min}<h_{i}<2r_{e}\sin\alpha ) \end{array}$$

with

(11) $$\theta_{2}=\{\begin{array}{c} {\text{cos}}^{-1}\left[\frac{h_{i}^{2}\;+\;r_{e}^{2}\;-\;{\left(r_{e}\;-\;h_{\text{min}}\right)}^{2}}{2r_{e}h_{i}}\right]\;\;\;\;\;\;\;\;\;\;\;\;\;\;\;\;\;\;\;\;0<h_{i}<r_{e}\text{cos}\left[{\text{sin}}^{\;-\;1}\frac{\left(r_{e}\;-\;h_{\text{min}}\right)\text{sin}\left(\frac{\pi }{2}\;-\;\alpha \right)}{r_{e}}\right]\\ \alpha +{\text{sin}}^{-1}\frac{\left(r_{e}\;-\;h_{i}\text{tan}\alpha \;-\;h_{\text{min}}\right)\;\times \;\text{sin}\left(\frac{\pi }{2}\;+\;\alpha \right)}{r_{e}}\;\;\;\;r_{e}\text{cos}\left[{\text{sin}}^{\;-\;1}\frac{\left(r_{e}-h_{\text{min}}\right)\text{sin}\left(\frac{\pi }{2}\;-\;\alpha \right)}{r_{e}}\right]\leq h_{i}\leq h_{\text{max}} \end{array}$$

and

(12) $$\theta_{3}=\pi -\cos^{-1}\frac{h_{i}^{2}+{\left(r_{e}-h_{\min}\right)}^{2}-r_{e}^{2}}{2h_{i}\left(r_{e}-h_{\min}\right)}$$

Further, the calculation of $A_{t}^{i}$ and $A_{p}^{i}$ at point C has to consider the clearance angle and the round cutting edge. The tool edge profile ($z_{e}^{ij}$) at the discrete point $x_{j}$ in the direction of $\varphi_{i}$ can be written as:

(13) $$z_{e}^{ij}=\{\begin{array}{c} -\sqrt{{r_{e}}^{2}-{\left(x_{j}-h_{i}\right)}^{2}}\;\;\;\;\;\;\;\;\;\;\;\;\;\;x_{j}>h_{i}-r_{e}\sin\alpha \\ -\tan\alpha \times \left(x_{j}-h_{i}\right)-\frac{r_{e}}{\cos\alpha }\;\;\;\;\;\;\;\;\;\;x_{j}\leq h_{i}-r_{e}\sin\alpha \end{array}$$

where α is the clearance angle of the milling tool edge. Point C, $A_{t}^{i}$ and $A_{p}^{i}$ can be written as:

(14) $$A_{t}^{i}=r_{e}^{2}\times \theta_{1}-\frac{h_{i}^{2}\tan\alpha }{2}-\frac{r_{e}\sin\theta_{1}\left(r_{e}-h_{i}\tan\alpha \right)}{2}+dh_{i}\;\;\;\;\;(2r_{e}\sin\alpha <h_{i}<h_{\max})$$

When $2r_{e}\sin\alpha <h_{i}<r_{e}\cos\left[\sin^{-1}\frac{\left(r_{e}-h_{\min}\right)\sin\left(\frac{\pi }{2}-\alpha \right)}{r_{e}}\right]$, $A_{p}^{i}$ can be computed using Equation (10), and when $r_{e}\cos\left[\sin^{-1}\frac{\left(r_{e}-h_{\min}\right)\sin\left(\frac{\pi }{2}-\alpha \right)}{r_{e}}\right]\leq h_{i}<h_{\max}$, $A_{p}^{i}$ can be computed as:

(15) $$\begin{array}{l} A_{p}^{i}=A_{t}^{i}-{\left(r_{e}-h_{\text{min}}\right)}^{2}\left(\frac{\pi }{2}+\alpha \right)-\frac{{\left(r_{e}-h_{\text{min}}\right)}^{2}\text{sin}\alpha \times \text{cos}\alpha }{2}\\ \;+\frac{{\left[h_{i}-\left(r_{e}-h_{\text{min}}\right)\text{sin}\alpha \right]}^{2}}{2}\text{tan}\alpha \;+\left(d-r_{e}\right)(h_{i}-h_{\text{min}})+r_{e}^{2}\times \theta_{2}\\ \;-\left(r_{e}-h_{\text{min}}\right)\left[h_{i}-\left(r_{e}-h_{\text{min}}\right)\text{sin}\alpha \right]\text{cos}\alpha \\ \;+\frac{r_{e}\text{sin}\theta_{1}}{2}\left[\left(r_{e}-h_{\text{min}}\right)\left(\text{cos}\alpha +\text{sin}\alpha \cdot \text{tan}\alpha \right)-h_{i}\text{tan}\alpha \right] \end{array}$$

${\mathrm{NSPA}}_{i}$ for point B and C can then be calculated by Equation (2). Thus, the surface uniformity can be simulated by calculating the change in the stochastic roughness of the cross-sectional direction, based on Equation (1).

Material elastic recovery has a much higher impact on average surface roughness than surface uniformity. Similarly, tool vibrations exist throughout the tool rotation trajectory in micro-milling, so even though forced tool vibrations can increase surface roughness as a whole, its influence on surface uniformity is not so obvious. As a consequence, elastic recovery and tool vibrations are neglected in the model.

To determine *k* and *n*, 100 sub-regions with equal width in the cross-sectional direction of the micro-grooves were sampled, and the surface roughness of each sub-region $Ra^{i}$ was measured. The stochastic roughness of each sub-region $Ra_{s}^{i}$ was then calculated by $Ra_{s}^{i}=Ra^{i}-E(Ra^{i})$, where $E(Ra^{i})$ is the mathematical expression of $Ra^{i}$. ${\mathrm{NSPA}}_{i}$ at each rotation angle $\varphi_{i}$ is then separately calculated based on Equation (2). Finally, *k* and *n* can be determined by the least square fit arithmetic.

## 4. Results and Discussion

### 4.1. Surface Uniformity

**Figure 4:**
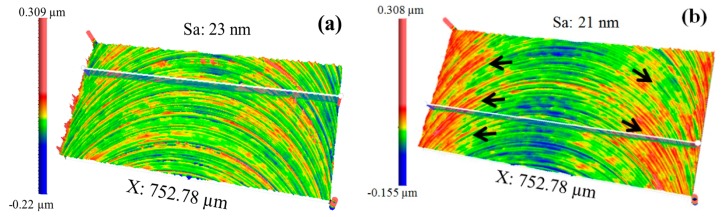
3D surface topographies for the feed rate at (**a**) 0.4 µm/flute and (**b**) 0.8 µm/flute.

The 3D surface topographies generated at different feed rates are compared in Figure 4a,b. It is clear that the surface topography machined at 0.4 µm/flute, as shown in Figure 4a, is more uniform than that machined at 0.8 µm/flute, as shown in Figure 4b. The non-uniform surface of 0.8 µm/flute is characterized by a rugged texture, distributed at both the up-milling and down-milling sides, as marked by the black arrows in Figure 4b. However, it is interesting to note that the surface roughness of 0.8 µm/flute is actually slightly lower than that of 0.4 µm/flute, measured at 21 nm Sa and 23 nm Sa, respectively. As a result, it is seen that the uniformity of micro-milled surfaces can be greatly changed by using different machining parameters, and the surface quality evaluation methods normally adopted, i.e., Sa or Ra, are not appropriate to the evaluation of surface uniformity.

**Figure 5:**
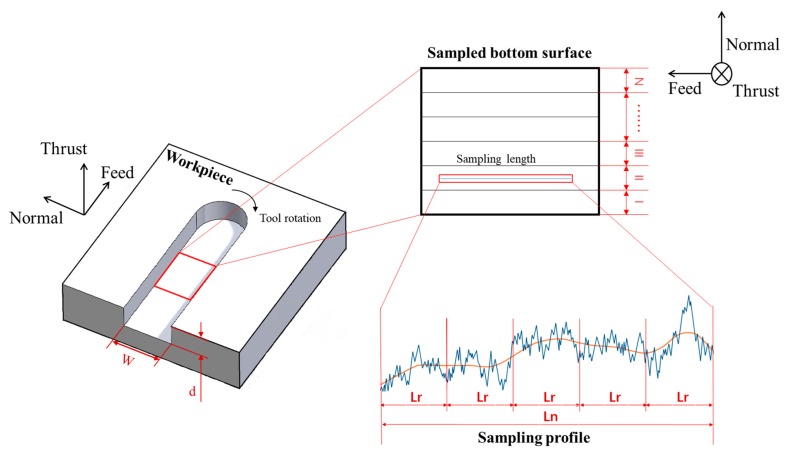
Model for calculating relative standard deviation of roughness (RSDS).

To achieve effective evaluation of the surface uniformity, a method, named the relative standard deviation of surface roughness (RSDS), is proposed in the present study. The model for calculating RSDS is schematically illustrated in Figure 5. A micro-milled groove is equally divided into N sub-regions in the normal direction. Then, the profile surface roughness of each sub-region is separately measured, before the RSDS is calculated. Specifically, RSDS is defined as the ratio of the standard deviation of the N profile surface roughness (σ) to the average surface roughness (µ), which can be expressed as:

(16) $$\mathrm{RSDS}=\frac{\sqrt{\frac{1}{N}{\left(N\times Ra_{i}-\sum_{i=1}^{N}Ra_{i}\right)}^{2}}}{\sum_{i=1}^{N}Ra_{i}}$$

where $Ra_{i}$ is the profile surface roughness for the *i*-th sub-region.

### 4.2. Effect of Machining Parameters

**Figure 6:**
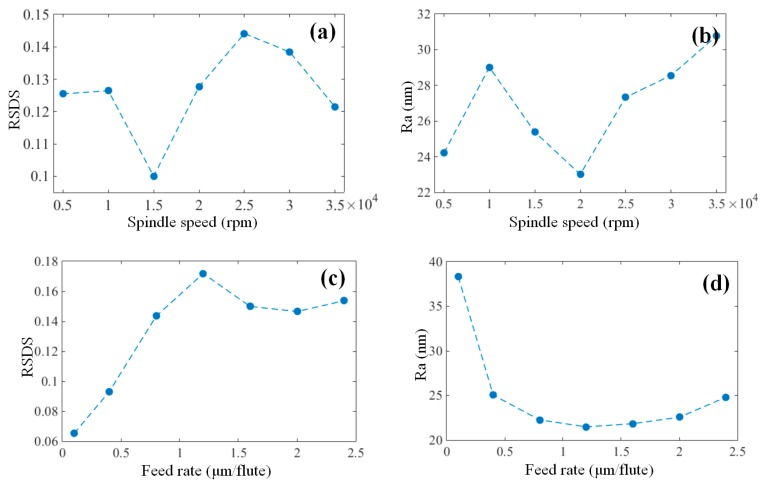

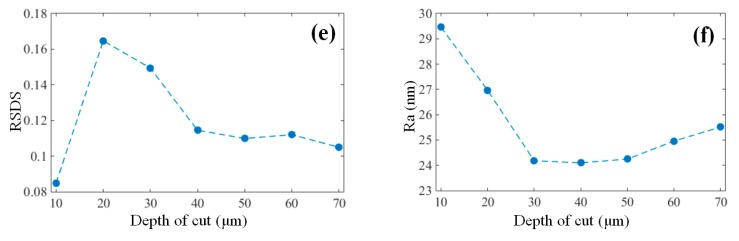
The trend of RSDS and Ra of the micro-milled grooves under different machining parameters with (**a**,**b**) spindle speed, (**c**,**d**) feed rate and (**e**,**f**) depth of cut.

The effects of the machining parameters on the surface uniformity and surface roughness are comparatively discussed in this section. As shown in Figure 6a,b, no obvious varying trend was observed for RSDS and Ra, as the spindle speed increased, indicating the small influence of the cutting speed on the surface quality. This was probably due to the utilization of the high precision air-static spindle in the experiment, which has good dynamic properties even under high rotational speeds.

In contrast, RSDS and Ra presented opposite variation trends, as the feed rate increased, as shown in Figure 6c,d. In other words, even though the surface roughness decreased as the feed rate increased, the surface uniformity decreased (the higher the RSDS values, the lower the surface uniformity) at the same time. The decreased roughness results from the reduction of the ploughing effect at higher feed rates. On the other hand, as the chip load in micro-milling was very small, a very slight increase in chip thickness from the groove sides to the center, as the tool rotates, could significantly reduce the level of ploughing. This then led to the acquisition of a lower roughness in the groove center compared to that on the sides. When the feed rate increased at a specific value, the chip thickness in the groove center increased at the same value, while the chip thickness near the groove sides remained unchanged and approached zero, thereby resulting in the deterioration of the surface non-uniformity as the feed rate increased.

Interestingly, as shown in Figure 6e,f, both RSDS and Ra decreased as the depth of cut increased, which indicates the improvement of the surface uniformity and surface quality at higher cutting depths. This is because increasing the depth of cut increased the tool-workpiece contact area, and accordingly reduced the ploughing effect. The lower RSDS value, with a depth of cut at 10 µm, resulted from severe ploughing without shearing at this stage, so the surface was quite uniform but had a high surface roughness, as shown in Figure 6f.

### 4.3. Variation of Cutting Mechanisms

**Figure 7:**
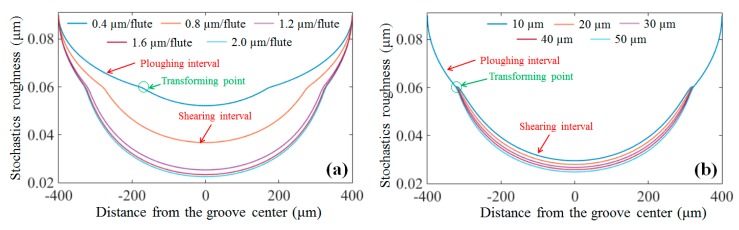
Stochastic roughness with different (**a**) feed rates and (**b**) cutting depths.

**Figure 8:**
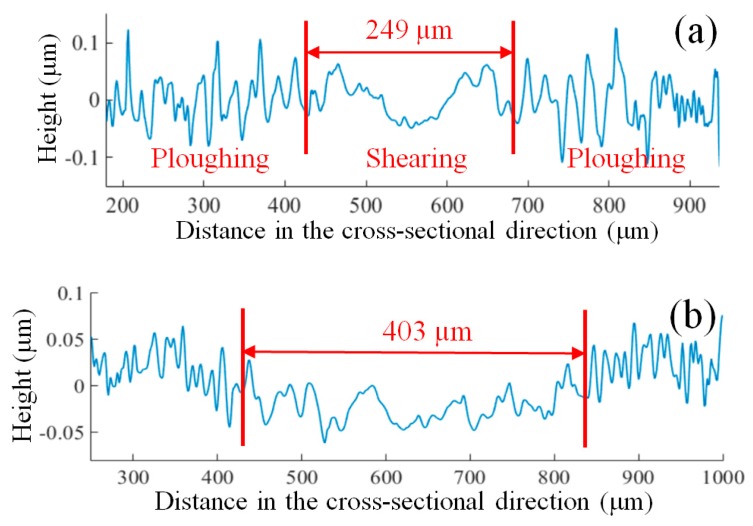

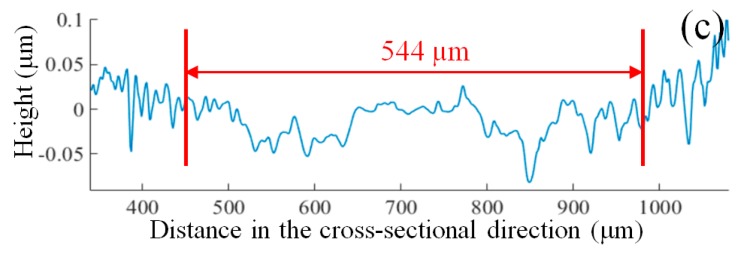
Cross-sectional profiles of the micro-grooves with a feed rate of (**a**) 0.4 µm/flute; (**b**) 0.8 µm/flute; and (**c**) 1.2 µm/flute.

The variation of the simulated stochastic roughness in the cross-sectional direction of the micro-milled grooves at different feed rates is shown in Figure 7a. The stochastic roughness in the groove center is much lower than that of the sides, and clear transformation points from ploughing to shearing are acquired. This transformation can well explain the generation of the non-uniform surfaces in micro-milling and shows that the increase in the chip thickness from the groove sides to the center reduces the effect of the ploughing on the surface quality near the groove center. Moreover, it is interesting to note that the length shearing interval increases as the feed rates increase, as shown in Figure 7a, which is highly consistent with the experimental results shown in Figure 8. Increasing the length of the shearing interval results in the deterioration of the surface uniformity as the feed rate increases, which is the major reason for the increasing trend of RSDS, as seen in Figure 6c.

**Figure 9:**
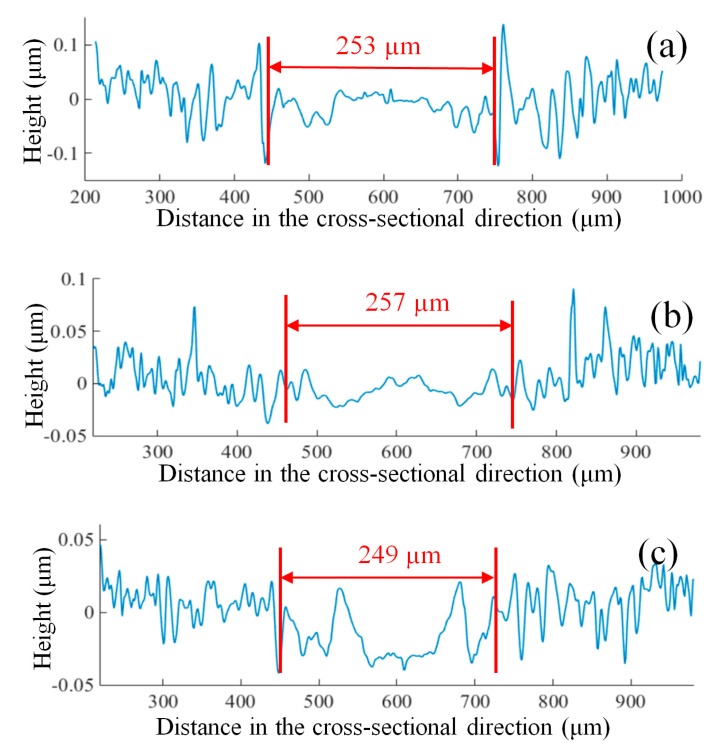
Cross-sectional profiles with a cutting depth of (**a**) 20 µm, (**b**) 30 µm and (**c**) 40 µm.

Nevertheless, the length of the shearing interval remains almost constant as the depth of cut increases for both the simulated and experimental results, as shown in Figure 7b and Figure 9, respectively. This is because the change in the cutting depth has no impact on the chip thickness within each tool rotation. However, increasing the cutting depth increases the contact area between the micro-milling tool and the workpiece, thereby reducing the effect of ploughing.

**Figure 10:**
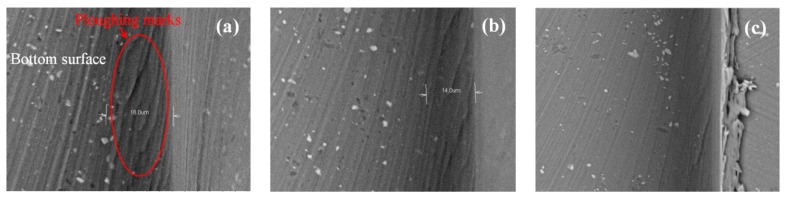
Surface topographies with a feed rate of (**a**) 0.8 µm/flute (**b**) 1.2 µm/flute (**c**) 1.6 µm/flute.

In addition, when the chip load exceeds a critical value, the influence of the ploughing effect on the surface uniformity can be neglected due to the dominance of the shearing mechanism at this stage. As shown in Figure 10a, clear rugged marks induced by ploughing can be observed at the boundary of the micro-groove, generated at a feed rate of 0.8 µm/flute. Nevertheless, the rugged marks are significantly reduced as the feed rate increases, as shown in Figure 10b,c, which validates the weakness of ploughing at higher feed rates.

### 4.4. Tool Wear

**Figure 11:**
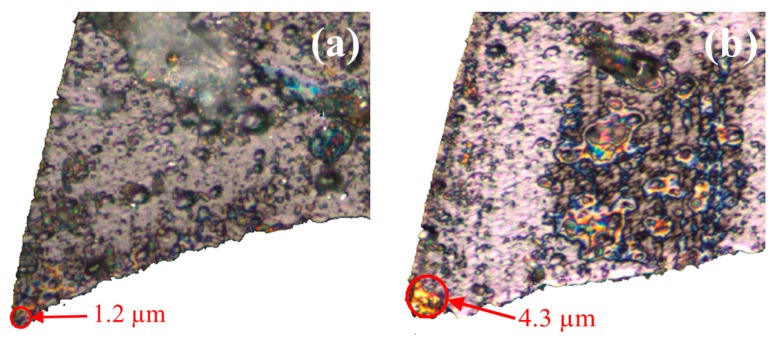
Optical images of the (**a**) new tool and (**b**) worn tool.

**Figure 12:**
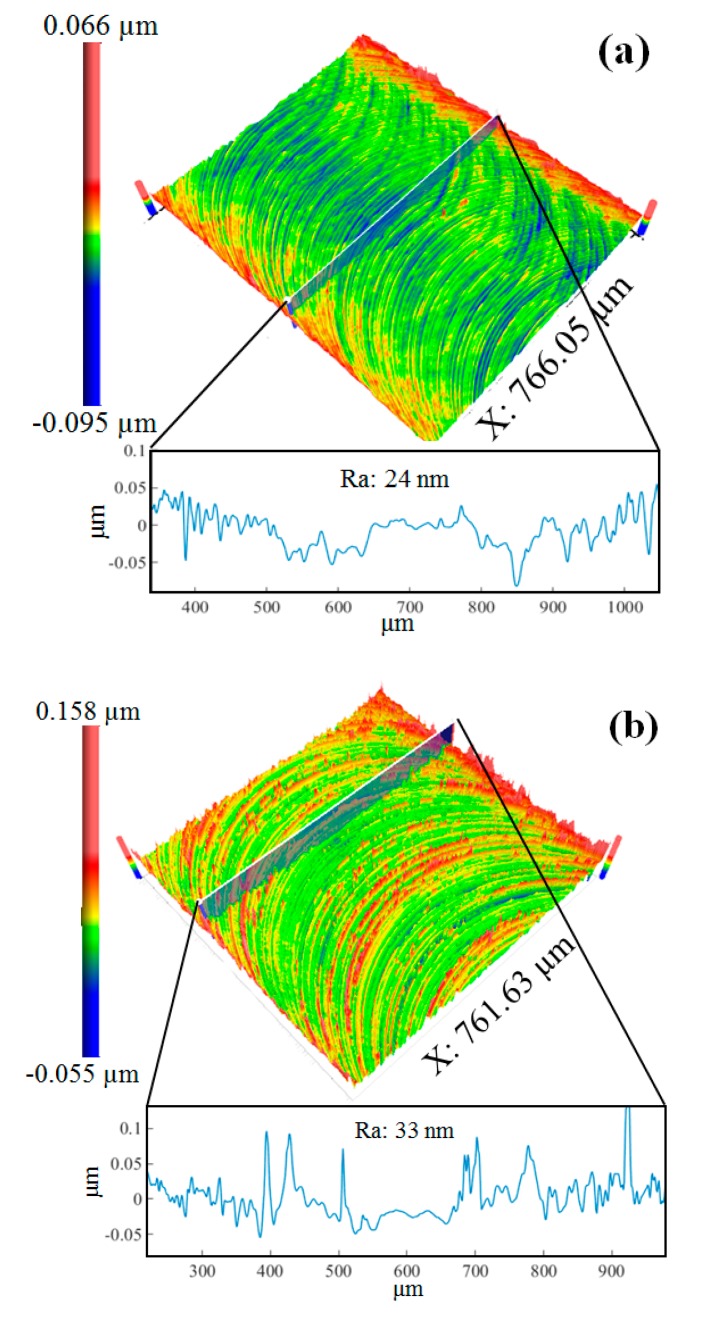
The 3D topography and the 2D profile of the micro-groove machined by (**a**) the new tool and (**b**) the worn tool.

As seen in Figure 11a, the tool edge radius of the new micro-milling tool is measured at 1.2 µm, and the edge was significantly blunted after dry machining 50 micro-grooves, with the tool edge radius increasing to 4.3 µm. The 3D surface topographies generated by the new tool and the worn tool, with a feed rate of 1.2 µm/flute and a depth of cut at 30 µm, are presented in Figure 12a,b. It is clearly seen that the 3D topography generated by the worn tool is more uniform compared to that of the new tool. Using on the proposed model, it is shown that increasing the tool edge radius can prolong the ploughing interval and shorten the shearing interval, so the surface topography generated by the worn tool is more uniform. However, the severe ploughing effect of the worn tool also leads to a highly rugged surface texture and increased surface roughness, from 24 nm to 33 nm in the cross-sectional direction of the micro-grooves, as shown in Figure 12a,b, respectively. Another reason for the high surface roughness induced by the worn tool is the increase in cutting force amplitudes and severe tool vibrations [23].

## 5. Conclusions

This paper presents a theoretical and experimental study on the effects of machining parameters and tool wear on surface uniformity in micro-milling. A mathematical model is proposed and experimentally evaluated to estimate the variation of the roughness in the cross-sectional direction of the micro-grooves. The key conclusions drawn are as follows:

(1) Compared with the Ra or Sa, the proposed evaluation method, named the relative standard deviation of roughness (RSDS), is more appropriate to evaluating the change in micro-milled surface uniformity with different machining parameters.
(2) Even though the surface roughness (Ra) decreases as the feed rate increases, when the feed rate is lower than 1.6 µm/flute, the surface uniformity decreases at the same time. This is due to the increased length of the shearing interval in the groove center.
(3) Both surface uniformity and surface quality improve as the depth of cut increases. This is due to the reduced ploughing effect induced by the increasing contact area between the tool edge and the material.
(4) The blunt tool edge enhances the micro-milled surface uniformity, but it also results in high surface roughness due to the severe ploughing effect under the worn tool.

## Figures and Tables

**Table 1:** Machining parameters.

Parameters	Group 1	Group 2	Group 3
Spindle speed (rpm)	5000–35,000	20,000	30,000
Feed rate (µm/flute)	1.2	0.1–2.5	0.8
Depth of cut (µm)	30	30	10–70

**Table 2:** Machining conditions.

The number of sampling points of surface roughness (N)	10
Tool runout length ($r_{o}$) (µm)	3
Tool runout angle (δ) (°)	50
Tool edge radius ($r_{e}$) (µm)	1.2
Tool clearance angle (α) (°)	10

## Nomenclature

$\left(x_{c}^{i},y_{c}^{i}\right)$	Coordinates of tool edge center in the direction of $\varphi_{i}$
$x_{j}$	The coordinate of the j-th point in feed direction
$\varphi_{i}$	The i-th tool rotation angle
$\gamma_{i}$	Instantaneous negative rake angle
$h_{i}$	The instantaneous uncut chip thickness in the direction of $\varphi_{i}$
$h_{\min}$	Minimum chip thickness
$h_{\max}$	Maximum uncut chip thickness
*Ra_i_*	The surface roughness of the *i*-th discrete region
µ	Average value of N discrete surface roughness
$r_{e}$	Tool edge radius
D	The diameter of the milling tool
N	The number of the discrete regions
$A_{t}^{i}\;\;\;\;\;\;\;\;\;\;$	The total volume of tool contact material in the direction of $\varphi_{i}$
$A_{s}^{i}$, $A_{p}^{i}$	The volume of sheared and ploughed material in the direction of $\varphi_{i}$
${\mathrm{NSPA}}_{i}$	The i-th normal specific ploughing amount
σ	Standard deviation of N discrete surface roughness
$n_{f}$	The ordinal number of tool flutes
$f_{t}$	Feed per tooth
d	Depth of cut
$z_{e}^{ij}$	The height of tool edge profile
N	The number of sampling points of surface roughness
*n*	Spindle rotation speed
α	Tool clearance angle
